# The Effect of Neighborhood Experiences on Positive Mental Health Among Community-Dwelling Older Adults

**DOI:** 10.1093/geroni/igaa057.1408

**Published:** 2020-12-16

**Authors:** Daniel R Y Gan, Grand H-L Cheng, Tze Pin Ng, John Chye Fung, Im Sik Cho

**Affiliations:** 1 Simon Fraser University, Vancouver, British Columbia, Canada; 2 The Open University of Hong Kong, Hong Kong, China; 3 National University of Singapore, Singapore, Singapore

## Abstract

Given reduced life spaces, the neighborhood often functions as a social venue for older adults. Yet how these everyday social spaces affect older adults’ psychosocial wellbeing remains largely unknown. Drawing on the GRP-CARE Survey data, this paper examined the relation between neighborhood experiences and positive mental health. Participants were 601 community-dwelling Singaporeans aged 50+ who lived in public housing neighborhoods. Neighborhood experiences were measured using the four-factorial, 16-item OpenX scale (Gan, Fung, Cho, 2019); positive mental health was measured using a six-factorial, 19-item scale (Vaingankar et al., 2011). Both scales have good psychometric properties and had been validated. Path analysis between relevant factors of both scales was conducted using Stata, within a theorized model of causation from neighborhood environment to social factors to psychosocial health. Age, education, ethnicity and sex were controlled for. Multiple linear regression analysis showed a strong, positive association between neighborhood experiences and mental health (p=0.000) even after controlling for personal traits (operationalized as depressive symptoms, GDS) in addition to sociodemographic variables. Path analysis showed that two distinct neighborhood health processes mediated this association. These were (1) the potential for a sense of community in the neighborhood improved emotional support, and (2) having better neighborly friendships improved interpersonal skills. These neighborhood health processes provide us with new lenses to understand older adults’ everyday experiences of their neighborhoods. Community-based interventions to improve older adults’ psychosocial wellbeing may be developed to facilitate these processes. Spatial and programmatic implications will be discussed in relation to age-friendly cities and communities (AFCC).

